# Perceived needs and experiences with healthcare services of women with spinal cord injury during pregnancy and childbirth — a qualitative content analysis of focus groups and individual interviews

**DOI:** 10.1186/s12913-015-0878-0

**Published:** 2015-06-16

**Authors:** Sue Bertschy, Szilvia Geyh, Jürgen Pannek, Thorsten Meyer

**Affiliations:** Swiss Paraplegic Research (SPF), Guido A. Zäch-Strasse 4, CH-6207 Nottwil, Switzerland; Department of Health Sciences and Health Policy, University of Lucerne and SPF, Nottwil, Switzerland; Swiss Paraplegic Center (SPZ), Guido A. Zäch-Strasse 2, CH-6207 Nottwil, Switzerland; Hannover Medical School, Institute for Epidemiology, Social Medicine and Health Systems Research, Carl-Neuberg-Str. 1, D-30625 Hannover, Germany

**Keywords:** Spinal cord injury, Sci, Pregnancy, Childbirth, Women, Health care services

## Abstract

**Background:**

Women after a spinal cord injury (SCI), who decide to get pregnant and to become mothers, have special health care service needs. This study aims to identify the perceived service needs of woman with SCI during pregnancy and childbirth in Switzerland and to reconstruct their experiences of healthcare service utilization based on their accounts.

**Methods:**

A qualitative content analysis based on focus groups and individual interviews was conducted. 17 mothers with SCI who had given birth following SCI within the past 15 years participated. The data were transcribed verbatim before content analyses were carried out. Primary data was collected from August 2012 to September 2013 at the Swiss Paraplegic Research Centre, Nottwil; the University of Lausanne and at the homes of the participants.

**Results:**

Mothers reported a broad spectrum of medical needs, including the need for access to improved integrated care. They also reported difficulties finding providers with knowledge of both paraplegiology (i.e. spinal cord medicine) and gynaecology. Mothers preferred using local health care services and regular birth hospitals, and reported receiving no additional benefit from the services of specialised SCI centres during pregnancy. A pre-existing provider-patient relationship was helpful for optimizing care processes.

**Conclusion:**

This study showed that pregnant women with SCI have various perceived healthcare needs and health care service use. Effective programs to improve these women’s access to integrated care during pregnancy and childbirth and policies requiring the provision of specific pregnancy information and pre-birth services are necessary.

## Background

A woman’s desire to bear children often does not disappear after she has sustained a spinal cord injury (SCI), and women hoping to become mothers after a SCI have special health care service needs [1, 2], including accessibility of health care facilities, adjustment of assistive devices, counselling and changes in medications. Several qualitative studies from the United States (US) [3–6] suggest that the needs of these women are often not fully met.

Although pregnancy outcomes seem to be favourable after SCI [7–9], women with disabilities may face problems during pregnancy and childbirth due to physical barriers, lack of specialised services, health care system issues, and communicational, informational and attitudinal barriers [10, 11]. Health care providers have also described difficulties in service provision for women with disabilities during pregnancy and childbirth from their perspectives [12–16]. The limited knowledge available about pregnancy and childbirth after SCI has been poorly disseminated to stakeholders, including women with SCI. Most of these women report that they did not receive adequate information before becoming pregnant [17].

Previous studies have shown that it is important to meet the needs of pregnant women with SCI to ensure optimal health of the mother and child, to ensure participation and quality of life, to ensure equity of service provision and to avoid discrimination [2, 10, 18–24].

The United Nation’s *Convention for the Rights of People with Disabilities* (Art. 25) emphasizes the right to best health care services and explicitly stresses the relevance of sexual and reproductive health [25]. The World Health Organization also launched a program to promote reproductive health among people with disabilities [21, 26]. In the context of the health care rights of people with disabilities, SCI can serve as a case in point illustrating the concerns of women with physical disabilities during pregnancy and childbirth.

However, research on reproductive health care services for women with SCI is scarce. Current reviews of the literature reveal a focus on physical and other barriers [27]. These studies also lament the disparities in obtaining reproductive health care services [10] among women with disabilities, including women with SCI, especially in North America. In addition, the research has documented health risks and secondary complications during pregnancy and childbirth in women with SCI [8, 22, 23, 28, 29]. A clinical practice guideline edited by a consortium for spinal cord medicine in the US summarized the existing medical evidence on fertility, birth control, pregnancy, labour, delivery and menopause in women with SCI [30]. However, the guideline depicts the professional perspective, and there is a lack of sufficient knowledge on health care needs from the perspectives of women with SCI who are pregnant or planning a pregnancy.

Therefore, an increased understanding of the use of health care services during pregnancy and childbirth after SCI is required to optimize available services [22, 31]. Several questions have arisen requiring answers, including: *Which services are needed, provided and used? Why and with which result? To what degree are the services tailored to the perceived needs of the pregnant women? What constitutes a good health care experience for pregnant women with SCI?*

The aims of this paper are to identify the perceived service needs of woman with SCI during pregnancy and childbirth in Switzerland and to reconstruct their experiences of healthcare service utilization based on their accounts.

## Methods

### Study design

In order to identify the perceived service needs of woman with SCI during pregnancy and childbirth and to reconstruct their experiences of healthcare service utilization we used an open, qualitative research design. Women’s accounts on their health care experiences were elicited in focus groups and, in addition, in individual interviews. Both data from focus groups and interviews were transcribed verbatim. The transcripts were analysed using qualitative content analysis [32], an inductive coding approach wide-spread in the German health research [33]. This approach was chosen because it is suitable to analyse how women interpret and make meaning from their health care experiences and to interpret their accounts in lights of potential unmet service needs.

### Participants

**Fig. 1:**
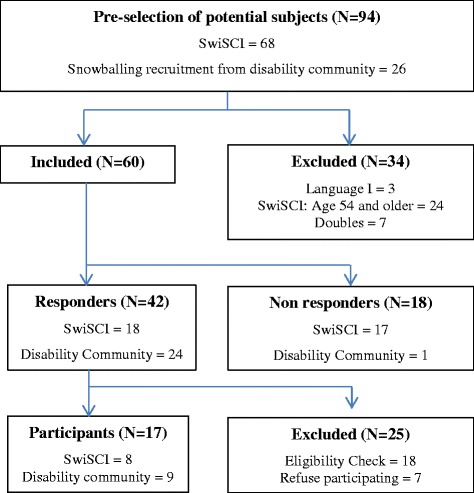
Recruitment of study participants

Participants characteristics were aligned with the Swiss spinal cord injury cohort study (SwiSCI) [34]. We included 18–55-year-old mothers diagnosed with traumatic or non-traumatic SCI, who were permanent residents in Switzerland and gave birth after the injury within the past 15 years. Individuals with congenital conditions leading to paraplegia or tetraplegia were excluded: spina bifida, sclerosis and amyotrophic lateral sclerosis, and Guillan-Barre’ syndrome. We used different pathways to recruit potential participants. Mothers were recruited through the Swiss Spinal Cord Injury Cohort (SwiSCI) [34] database and through snowball sampling in the disability community [35]. Both lists were matched and all 94 names were cross-checked. We excluded seven duplicates, three Italian speaking mothers and 24 women over 55 years of age. The remaining 60 eligible women received information about the study aims, the study procedures and a response card to indicate their willingness to participate. The response rate was 70 % (42 women). These respondents were contacted by telephone for a second eligibility check. Of these mothers, 18 did not meet the eligibility criteria and seven declined to participate. Seventeen mothers expressed interest in taking part in the study, representing a final response rate of 28 %. The recruitment of the study participants is illustrated in Fig. 1.

### Data collection

Focus groups and individual interviews were conducted. The original aim was to assemble the women for group discussions; however, many women asked for individual interviews because of organizational, personal and language (German/French) concerns. Four focus groups and five individual interview sessions were held. The interviews were moderated by the principle investigator, supported by one assistant. The principle investigator was a wheelchair-user woman who encouraged the women to describe their experiences with health care providers and services. She sought to provide an open atmosphere to allow participants to share personal experiences. The pilot-test revealed that more serious discussions typically emerged after about one hour of interaction. Therefore we chose small groups to activate the discussions [36]. Focus groups took place at the Swiss Paraplegic Research centre in Nottwil, at the University of Lausanne, and individual interviews were done at the participants’ homes. The focus group sessions were approximately 120–180 min, and the individual interviews were approximately 45–60 min. The interviews were semi-structured and guided by previously developed key questions [37]. The key questions were formulated based on a theoretical model of health services Andersen’s [38], review of the literature and expert knowledge. The interview guideline was pilot-tested with a focus group of four mothers who did not match the eligibility criteria and who had given birth more than 15 years prior.

The participants were asked about their experiences during pregnancy and childbirth. Experiences included details regarding professional support providers, the types of care these professional provided, which providers offered care for which problems, whether the women felt to have received appropriate support for their health care needs, their satisfaction with the information received and suggestions for improved care in the future.

Participants received a demographic questionnaire and an informed consent form, which they completed and signed before the interview. On two occasions, two different mothers brought their children to the focus group session. The study drop-out rate was zero. The interview sessions were held in the Swiss German or French languages, were electronically voice-recorded and were transcribed with f4 software for data analysis. The study was approved by the ethics committee of the Canton Lucerne on 27 April 2012 (project number 12027).

### Analyses

#### Theoretical model

**Fig. 2:**
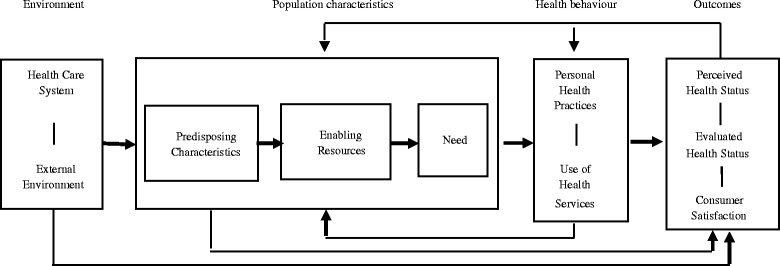
Model of health care services utilisation [38] p. 8

The study and qualitative content analyses were informed by the behavioural model of health services utilisation by Andersen [38], which is one of the most widely used frameworks in health services research regarding service utilisation [39] (Fig. 2).

The model comprises four domains: environmental factors, population characteristics, health behaviour and health outcomes. Environmental factors related to policy and health system determinants that describe the context of service use. Population characteristics include a number of predisposing factors that determine the use of health services. These include the individual’s predisposition to obtain services and the perceived or normative need for these services. Health behaviour is expressed within the contexts of individual health practices and the use of health services. Health outcome dimensions describe perceived and evaluated health status and consumer satisfaction.

#### Analysis process

We used content analysis [32, 40] to structure the transcribed interview materials. Our approach was to utilise the women’s reports of health service experiences to determine service needs and use of services. Our conceptual approach was based on Andersen’s behavioural model. We created a coding scheme with main categories derived deductively from the Andersen model [38]. The main categories were 1) women’s perceived health needs and 2) the health services used. The sub-categories were derived inductively from the data. Inductive coding was used to ensure that our analyses comprehensively represented the content communicated by the participants. The categories were ordered within a taxonomy, where each category received a specific definition. The codes were developed successively and were revised or deleted and new codes added as the analyses proceeded.

### Rigor

A number of steps were taken to ensure the trustworthiness of this qualitative study. *Confirmability* [41] was ensured by including various researchers during the analysis process. Two researchers participated in developing the codes, conducting the coding and the taxonomy. The first author completed the initial coding and met with the second author regularly to discuss emergent themes and alternate interpretations to guide further analyses. The main coder completed all transcripts and compared the results in determined sections with the co-coder until a common coding was established. Thoughts and consensus solutions were summarized in a separate report sheet.

*Credibility* [41] was enhanced through the involvement of all co-authors at different stages of the study. The main investigator and the second author formulated the interview questions, developed the coding scheme and analysed and interpreted the data. During these steps, the researchers met regularly with the senior author. The other contributor conducted a critical revision of the manuscript for important intellectual content. The research team members possessed expertise in health service research, psychology, medicine and living with an SCI.

*Transferability* [41] of the results was enhanced by the inclusion of women with different lesions and education levels who had given birth during different periods within the 15 years prior, therefore covering the bandwidth of the study period. Within the results description, we attempted to display the different experiences in their heterogeneity.

## Results

### Participants’ characteristics

The participants included 17 mothers, including one pregnant woman with SCI^i^. The average age at the time of delivery of the first child following SCI was 34 years. All the women were married or in stable relationships at the time of their pregnancies. They had high levels of education and most were employed. Nearly half of the women worked in health care contexts as nurses, nutritionists, pharmacists, psychologists or life coaches for disabled people. Most were rehabilitated in SCI centres if their injuries occurred in adulthood. Those injured as children were rehabilitated in children’s hospitals. All names in the manuscript are pseudonyms and stated in Table 1 of participants’ characteristics.

**Table 1:** Participant's characteristics

Pseudonym	Age by interview	Marital status by interview	Number of children before SCI after SCI		TSI (y) at birth of 1st child	SCI level		Education	Education years	Working	Interview
Emma	39	married	0	1	19	Paraplegia	incomplete	Apprenticeship/Training	15	yes	FG 1
Anna	41	married	0	1	22	Paraplegia	complete	Apprenticeship/Training	12	yes	FG 1
Lea	32	partnership	0	0	pregnant	Tetraplegia	incomplete	Undergrad or college degree	14	yes	FG 1
Emily	50	married	1	1	7	Paraplegia	complete	Post graduate	23	yes	FG 2
Lilly	49	married	0	2	14	Paraplegia	incomplete	Undergrad or college degree	19	yes	FG 2
Sara	44	married	0	2	3	Paraplegia	complete	Apprenticeship/Training	12	yes	FG 2
Marie	45	married	0	2	7	Tetraplegia	complete	Undergrad or college degree	17	yes	FG 2
Amelie	34	married	0	1	11	Paraplegia	incomplete	Apprenticeship/Training	12	no	FG 3
Lina	38	married	0	2	17	Paraplegia	complete	Post graduate	18	yes	FG 3
Maya	45	divorced	0	1	5	Paraplegia	complete	High school	8	yes	FG 3
Claudia	47	married	0	2	15	Paraplegia	complete	Apprenticeship/Training	12	no	EI 5
Rachel	43	partnership	1	1	2	Paraplegia	incomplete	Undergrad or college level	17	yes	EI 4
Laura	32	single	0	1	2	Paraplegia	incomplete	Undergrad or college degree	15	no	EI 1
Margrit	45	married	0	2	13	Tetraplegia	incomplete	Apprenticeship/Training	13	yes	FG 4
Jeannine	30	married	0	2	24	Paraplegia	complete	Undergrad or college degree	21	yes	FG 4
Monika	48	partnership	0	1	19	Paraplegia	incomplete	Undergrad or college degree	19	yes	EI 2
Kathrin	44	single	0	1	15	Tetraplegia	incomplete	Apprenticeship/Training	17	yes	EI 3

*TSI*, time since injury

### Perceived service needs during pregnancy

We learned from the participating women that pregnancy after SCI required extended and tailored health care services. The women’s reports about their perceived needs were grouped into six categories: (1) perceived need for information about SCI and pregnancy; (2) perceived need for specific health professionals’ expertise; (3) perceived need for medical treatment; (4) perceived need for access to and availability of care facilities; (5) perceived need for specific supplies and equipment and (6) perceived need for improved integration of care.

#### Perceived need for information about SCI and pregnancy

The participating mothers reported having had questions regarding the interdependence of SCI and pregnancy. *‘What is different in my situation?’* was the driving question behind their quests for information.

*Besides medical questions, I had a lot of practical questions, like ‘Do I feel the child at all?’ —Laura*

*During my first appointment with the gynaecologist, I asked him if he had other patients like me before and if he knew the process. He told me: ‘No not at all.’ He said he could find out, but that he thought there was no problem. Then I said, ‘Yeah, but how am I going to give birth? And what will happen?’ I had a thousand questions besides all the standard questions of a standard pregnancy. Then he told me: ‘Well, we will see. I think you’re not a high-risk pregnancy. ‘He said:’ We’ll frame it as a normal pregnancy, and if you need monthly controls, we will do that, no problem.’ So, that was strange.—Jeannine*

#### Perceived need for specific health professionals’ expertise

Women reported having had a desire for providers with obstetrical as well as paraplegiological expertise. The lack of provider knowledge about the comprehensive needs of women with SCI during pregnancy spanned a range of themes. How to treat SCI-related conditions, how to distinguish between SCI-related and non-SCI-related conditions and how to evaluate treatments were perceived as difficult to handle.

*… I prefer a good gynaecologist who reads a little in the SCI literature. —Anna*

*What profit is paraplegiological knowledge if the doctor has no idea about neonatology? —Amelie*

*That was principally the most difficult question: Where are the knowledgeable people? Where are the top people? Only through asking friends I came to learn of this gynaecologist in the area. —Laura*

An existing relationship with a provider with knowledge of the SCI history was perceived by the women as helpful and was presented as a key issue in provider trust and continuance of care with the provider.

*My doctor said he would be confident in treating me. I was glad I did not had to change providers because we knew each other already and I trusted him. —Anna*

The education programs (e.g. pregnancy or other preparatory birth courses) were seen as inadequate. The women reported that the courses were not tailored to their needs and, therefore, were not utilised.

*It bothered me to go. I did not want to go… I was not interested in these types of courses. —Margrit*

*My doctor told me that it was useless for me because I would not give birth in a natural way. —Jeannine*

#### Perceived need for medical treatment

Women required medical treatment for issues with health and functioning. Their pregnancies exacerbated pre-existing functional difficulties, such as urinary tract infections (UTIs), spasticity and bowel problems. Pregnancy-related problems, including preeclampsia, pre- or postpartum symptoms or complaints e.g., signs of pre-term delivery, pre-term gravidity pain before calculated birth time and/or antepartum bleeding, post-term bleeding, infection and/or pain led to needs for medical treatments.

*I also had problems with the bowel, but the bladder was probably worse, I think. I noticed the difference with the bladder and the bowel was more constipated. — Rachel*

*I used self-medication, but always consulted my doctor [general practitioner] first, since I knew immediately when I had one [bladder infection]. —Margrit*

#### Perceived need for access to and availability of services

Participating women reported having had different experiences with the availability and accessibility of facilities, providers and educational programs. Women also revealed the special problem of not being able to identify or find SCI-knowledgeable providers or institutions. A woman described her difficulties finding a gynaecologist:

*…I’m always afraid when I have to look for a provider… there is no elevator, no parking and heavy doors. —Emma*

*…we looked for a gynaecologist who had a wheelchair-accessible practice, which is in many places not the case. —Isabel*

*I completely gave up looking for a knowledgeable provider. I had maybe not that much pressure because I had the feeling that I could do it by my own. Then I also did not insist too long searching for this one [gynaecologist with expertise in SCI]. There was definitely nobody who somehow had an idea about the topic or to provide information. I would not swear, but I could not remember having talked with anyone. —Lilly*

#### Perceived need for specific supplies and equipment

The women reported that inappropriately equipped facilities made examinations, diagnostics and hospital stays difficult and that very few facilities had the necessary equipment.

*It would certainly have been good to have a place where the whole infrastructure was available: an examination chair, enough space and rooms to fit our needs. —Laura*

*A lot of important equipment and tools were missing in the clinic. My husband had to bring almost our whole household to my room.— Marie*

For intimacy and privacy reasons, the women reported having preferred to stay alone rather than to share a room with able-bodied mothers.

*Due to my specific needs.... I found it embarrassing to explain to my room neighbour how my intimate care works. — Lina*

#### Perceived need for improved integration of care

Poor integrated care influenced women’s satisfaction with the care process. Since experts with holistic knowledge were unavailable, the women felt responsible to find knowledgeable providers for their care needs, which was inconvenient and disappointing to them.

*I would have appreciated a gynaecologist who had more experience with patients with SCI, who knew more about the bladder, all the hormones and bowel management. I sometimes ran desperately from one place to another… looking for a place where I could ask my questions and receiving answers… but not being able to consult the neurologists and the urologists and so on. But maybe that’s our destiny… that we will always have to see different providers. —Laura*

In addition, if participating women described different health behaviours, the need for comprehensive and continuous care as it would be provided with interdisciplinary provider teams was present. Some women would have appreciated to receive services from one site. The other group called for centralized or cooperative support services. Important for both groups was that their medical concerns were taken seriously.

*They [hospital staff] did not talk to each other. In the hospital of my home city, they filled in my medical chart with whatever they wanted. Every time, I had to re-explain my story and all my problems related to my pregnancy. —Jeannine*

#### Which health services were used?

The use of health care services was reported based on the involved health professional groups and facilities and the perceived degrees of service usage. The women’s experiences with health services differed in service choice, service variety and the number of services used.

#### Visited health professionals

Several women said that as soon as they believed they were pregnant, they felt an urgency to make appointments with their gynaecologists.

*As I realized that I was pregnant, immediately I wanted to visit my gynaecologist. He said I should come only after 5 weeks, because before then we would not see anything anyway. —Amelie*

In cases where the pregnancy course was uncomplicated, the women did not require additional medical care or monitoring. Regular gynaecological pregnancy monitoring included blood analyses, blood pressure monitoring, vaginal examinations, urine samples and foetal heart rate monitoring. The women reported having visited various health care providers, including general physicians, emergency physicians, urologists, neurologists and internists, when the pregnancy course was complicated by issues, such as hypertension, cardiovascular problems, UTIs, pneumonia or obstetric concerns. For treating specific SCI-related symptoms, women initially consulted their gynaecologists and, in some cases, a paraplegiologist or urologist with SCI knowledge. Latter consultations occurred in SCI outpatient settings. Women with incomplete lesions reported re-experiencing moments of anxiety and fear of functional limitations for which they required psychological counselling. One woman described her fear of re-experiencing loss of sensation in her legs:

*I can move [incomplete paralysis], so it was only psychological for me to go through that experience [epidural anaesthesia] again of losing the feelings, the sensation and movement, and not knowing if it would come back again. … Every week I saw a psychologist and talked about how I felt. I did some relaxation and breathing exercises and just… it was just a session for me to say these are my worries, this is how I feel, and he had good advice for me; this really helped. —Rachel*

The women also reported using complementary and alternative medicine services, such as naturopathy. One woman engaged a professional support person:

*It was important to me to have a person outside of us who knew my specific needs, too. I hired a woman as coach to support us. My husband told her things like, ‘my wife cannot lay on a hard surface’ and things like that. She was present during my birth. That gave me confidence. —Lilly*

Most of the expectant women found the support of experienced mothers helpful. They discussed personal issues, such as increasing belly size, incontinence or partnership issues, as well as which service facilities might be most helpful to address specific problems. The women reported that these encounters helped them become more comfortable with their pregnancies and reduced their fears about the upcoming deliveries. A woman described her appreciation:

*…what does it actually mean being pregnant and to give birth? What is possible? Those were my relevant questions. So the greatest help was certainly Monica [experienced mother], with her own experiences and her knowledge about where to get information. —Laura*

#### Consulted facilities

The women expressed desires for detailed information about pregnancy and SCI. In their attempts to meet these needs, the expectant mothers reported having consulted the Internet or having contacted SCI rehabilitation centres. Their contacts with the centres left the women feeling frustrated because their expectations were not met. One woman explained:

*I’m so excited about the bundled knowledge in this SCI centre. I think that’s such a good way to care for this population, as it takes place there. But the pregnancy area has been excluded, and that was very disappointing for me. —Lilly*

*I researched a little bit on the Internet. But even there, there is very, very poor information, almost nothing. —Lina*

Only one mother described having received a consultation hour with an external consultant gynaecologist in an SCI institution.

*She [gynaecologist] comes to the SCI centre [X] every month or couple of months to see women who would like to have children and to discuss any problems or any issues. I wanted to see her first, because I had some questions about what it meant —would it be a problem to have a child after the SCI —and to get some advice. —Rachel*

Inpatient stays in university or regional hospitals occurred mostly in emergency situations, such as those involving bladder problems with severe complications, pre-delivery symptoms or symptoms after a fall. In emergency settings, women described different types of difficulties in care continuity and reported lacks of efficiency compared to outpatient settings. The women reported that most treatment difficulties were encountered in bladder and anaesthesia complications. From the women’s perspectives, unfamiliarity in treating patients with a SCI led to inefficient treatments, unnecessary diagnostic tests and some unsafe treatment choices.

*I would like to mention that I was completely satisfied with my gynaecologist. But the fact that I had been in the hospital afterwards, and the assistant doctors were visiting me there… […] I remember a few scenes where they over-examined me. As my breasts were under the computer tomographer, I almost dismissed myself from the hospital. I started to argue with the doctor, because I felt this was leading nowhere. —Lina*

The women expressed different ideas of what a birth hospital should offer for women with SCI. Those with uncomplicated pregnancy courses tended to use birth hospitals in their home areas, while women with difficult pregnancy courses or with higher health literacy looked for well-reputed, nearby regional or university hospitals with neonatal units.

*…more important for me was that, if then something would have happened, they could take the baby and go only down the street and not drive through half of the city to bring the baby to the hospital with neonatology. —Anna*

Beside the available medical facilities for giving birth, the women reported having requested alternative options and described particular dissatisfaction with birthing houses. None of the participants gave birth in such an institution. Some of the women reported feeling somewhat unwelcomed in these institutions and described obvious apprehensiveness among the providers towards handling the pregnancy of a woman with an SCI. One woman described:

*I just wanted to have a look at this option (birthing centre) and they said that if the child remained hung with his shoulders during birth, I would have to immediately get out of the bathtub and transfer quickly to the birthing chair. If people like me needed help getting out of the bathtub —because I could not do it alone —that would be complicated. Also, they said you do not know how far the birth process is. If the head is already coming out, then I could not sit on the wheelchair and they would have to carry me over. That would be a risk. —Lea*

Some of the women with an incomplete lesion made use of the possibility to learn pre-birth pelvic floor exercises and a woman after child loss was grateful to visit a special remission course. However, some of the women, especially with a complete lesion were unconcerned, because a caesarean section was planned or they thought the courses were not tailored to their needs.

*I’m in a remission course for women after child loss. The exchange is different for women in difficult situations. —Laura*

*The pelvic floor exercises that were shown in the first course, I certainly could not do them (laughs). Yeah, I didn’t see the purpose of continuing the birth preparation course once it was clear that I would have a caesarean section. I did not need more breathing exercises (laughs). —Claudia*

#### Perceived degree of health services utilisation

Women judged their impressions of the pregnancies of able-bodied women in relation to their assumptions about these and their own pregnancies, and reported experiencing care very differently. The women with SCI reported having felt that they were seen more often by their gynaecologists, especially by the end of the third trimester.

*I saw my gynaecologist every 2 weeks and I had an ultrasound, which is not the case for all women. Given my situation, he acted very paternalistic. —Monica*

*In the beginning, I had a 6-week interval, and now I’m beginning the 7th month and we see each other every fifth week for controls. This is certainly something that is probably different for other women, unless they have a risky pregnancy. —Lea*

However, some women reported having felt that their care experiences were equal to those of able-bodied women.

*I think that I had fewer consultations than my other girlfriends who had been with another doctor. Today’s consultations are handled different. My doctor was experienced. — Lina*

Women reported that they were required to visit different providers to solve their health problems. They also mentioned that they were more likely hospitalized when the complications were not clear and that their hospital stays were longer.

*After being declared as a’risk pregnancy’ — I do not know what this word means — but risk means different. So I was treated differently. I think that’s why I stayed 10 days in the hospital. In general, it is rare that somebody has to stay 10 days there. —Kathrin*

## Discussion

This study sheds light on subjective service needs and the utilization of health services during pregnancy and childbirth of women with SCI in Switzerland.

Previous medical, disability and health service studies in other countries [8, 9, 11, 23, 29] have shown that pregnant women with SCI develop specific health care needs and face challenges obtaining services and specific treatments. Medical studies [8, 29, 7] have identified needs related to body functions, such as UTIs, autonomic dysfunction, skin breakdown and respiratory complications, whereas other studies [9, 11, 13] have reported environmental, structural and procedural needs (e.g. architectonical barriers, unfamiliar providers or no coordination of care). This study identified the full bandwidth of health care needs of women during pregnancy and childbirth.

Reproductive health services are in Switzerland for women sufficiently accessible and available. The density of gynaecologists is lower in the countryside than in urban areas, general practitioners and family doctors overtake also the obstetric part and cover a wider range of activities than their urban counterparts [42]. Though, the obstetric health service supply in Switzerland is satisfactory for able-bodied pregnant women [43]. There seems to be no research examining the accessibility to health services for disabled persons in Switzerland. In this study, women with SCI appear to be impacted by environmental barriers to health services during the perinatal phase. Access to public buildings (e.g., hospitals, schools, public buildings) is regulated in Switzerland since 2002 [44]. This law is only applicable to newly built or renovated public buildings and regulates access to and not the accommodation in facilities and does not include private buildings (e.g., private practices). Therefore a comfortable utilization for women with a disability of those facilities is not guaranteed which is also reflected in our results.

The women strongly emphasized their desires for expanded availability of providers with both pregnancy and SCI knowledge to provide wider service choices. Although this may not be possible in all settings, particularly in more rural areas, increasing the number of knowledgeable providers may improve these women’s likelihood of seeking needed care.

In Switzerland 94 % of the women give birth in a medical facility (hospital) and 6 % choose a birthing house or a birth at home as alternative solution. Traditionally no doctor is present in birthing houses and the birth is entirely handled by the midwife and the woman. The study results showed that women who were interested in this option were disappointed through the health professionals’ attitude to preferably refuse care for disabled women.

Participating women preferred to conduct the perinatal consultations in their home areas and only a few women requested counselling mainly for medication issues from specialised SCI settings. This could mean that those women who only used services in their home areas may not have known about or available counselling services in specialised SCI settings. Advertised and specialised and pregnancy counselling, as described in Tebbet’s work [1], may increase women’s self-confidence during pregnancy and reduce their dissatisfaction with the services offered at SCI centres.

The women communicated an elevated need for information about greater risks for secondary complications, pre-term births and caesarean deliveries, as reported in prior literature [45–47], which was also substantiated in this study. These perceptions highlight the challenges women may face when seeking needed services and being cared for by unfamiliar providers. These women’s perspectives also point to the critical need for more comprehensive services. Related to this need is the necessity for interdisciplinary cooperative networking not only for pregnant women with SCI but also for women with SCI planning to have children. Health care seeking could be addressed through regionally designated health professionals trained in providing perinatal care for these patients. In addition, a medical hotline could improve the effectiveness of care, and exchanges with experienced mothers may provide additional guidance. This exchange of experience could be provided in the form of a website for women with SCI, such as the Swedish project 'sciparenting.com' [48], which aims to provide information and knowledge about fertility, pregnancy, childbirth and parenting for persons with SCI. Tebbet [1] highlighted the positive experiences among women who participated in peer mentoring. Our study results also underlined this positive exchange, for which women reported to have a major impact in reducing anxiety and fear.

Lack of pre-birth counselling during pregnancy has also been identified in previous research [3, 49]. Burns [12] assumed in his survey that, with appropriate follow-up and care, women with SCI could be expected to maintain good gynaecologic health and deliver healthy children with minimal complications. However, the women in our study reported low rates of pre-birth education, showing the great need for pre-birth plans coordinating all involved parties. We suggest to include in this plan a checklist with information for women at each stage of pregnancy: a medical chart with information about medication and bladder management (facilitates caesarean), and X-rays of the spinal cord, if available (important for administrating anaesthesia).

It was interesting to note that the participating women tended to possess distinctive preconditions, which in some cases facilitated access to health services. Participating women tended to be highly educated, living in stable relationships, were socially integrated and seemed to be well informed about health issues. The question arises, then, if the women with SCI who decide to become mothers are different from women with SCI who do not desire to have children. This question should be further investigated in relation to epidemiological predictors.

Balancing SCI health care needs with available supply developed for able-bodied pregnant women may be a source of tension for some gynaecologists and SCI health care providers. The lacks of comprehensive knowledge, cooperation and regulations may also cause confusion, as specific guidelines in Switzerland have not been implemented in one field. Tailored information for women and providers should emphasize the availability of services to prevent mistreatment, under-treatment and over-treatment.

During the pregnancy course women have general or special medical needs. Study participants desired a medical treatment that took their special needs into account and at the same time they wished to be treated on the personal level in the professional-patient relationship as any other woman, i.e. without being discriminated on the grounds of their disability.

Women mentioned several medical complications, interestingly they did not describe or named the phenomena of autonomic dysreflexia, which is a common complication during the pregnancy course and birth process. We would like to point out, that this study reports the subjective perspective of the women how they experienced their need and use of healthcare. By not mentioning some for the reader evident or necessary medical needs doesn’t mean that they are not important. Contrary, this may uncover the existing gaps and missing medical knowledge which should be transferred to the women.

### Study limitations

Our findings are based on a self-selected sample; therefore, there is question as to whether the results reflect a selection bias in participant characteristics. As we included mothers with different lesion levels, paraplegia as well as tetraplegia, our results relate to women with SCI of childbearing age. Due to the different natures (medical and social) of other disabilities, however, we cannot be certain of a broader transferability of the findings to other disability groups.

The rather unusually small number of participants within the focus groups was, in our experience, not obstructive for obtaining responses. Rather, it allowed a relaxed and intimate interaction of respondents and stimulated a rich discussion including new and valuable thoughts and ideas. The participants in the individual interviews had more difficulties talking openly and required more time and empathy from the interviewer [36]. It has been reported that persons feel more open to share personal experience in homogenous group discussions compared to individual interviews.

Due to the differences in social welfare systems and medical health systems, it might be problematic to compare our results with those of other countries. Therefore, comparisons between countries focusing on the factors influencing service use would be beneficial. Interestingly, despite the differences between the social and medical systems of the countries, we did observe results compatible with the international literature.

Based on the aims of our study we used the Andersen model as a frame of reference to guide our interview questions and analysis. However, this approach might have influenced our openness to experiences women made during pregnancy and childbirth.

The data were analysed with a qualitative approach (a summative evaluative approach was not applicable). We had to rely on the openness and memories of the mothers for data collection in these retrospective accounts. Therefore, in the future, a prospective study with a follow-up of women with SCI at child bearing age or who are pregnant would be helpful.

## Conclusion

This study highlights several barriers that women with SCI face during pregnancy and childbirth. The existing health care services are far from being tailored to meet the needs and expectations of these mothers, and further improvements in both policy and practice are necessary to provide better health care to these women. Policy should provide a framework for health care providers that would allow them to most effectively meet the women’s needs. There is a high demand for specialised antenatal services that are more client centred, offering specific information, peer support or increased accessibility and availability of services. Further research is recommended to understand which factors most influence service use, to characterize the women with SCI who choose to become mothers and to examine the providers’ perspectives on accompanying women with SCI through pregnancy.

### Endnotes

^i^Due to the small number of participants who meet the inclusion criteria, we included in the study a woman pregnant in the third trimester who had experiences with the care system.

## Abbreviations

SCI: Spinal Cord Injury
US: United States
SwiSCI: Swiss Spinal Cord Injury Cohort Study
UTIs: Urinary Tract Infections
TSI: Time Since Injury
